# Charleston Medical Journal and Review

**Published:** 1873-04

**Authors:** 


*Charleston Medical Journal and Review.*

The first number of the new series of this journal has been received. It is issued quarterly, and edited by F. Peyre Porcher, M.D., and B. A. Kinlock, M.D. The high scientific reputation of the editors is the very best assurance to the profession of the future usefulness of this journal, and we cordially commend it to our readers as one every way worthy of their confidence and support. Subscription price, three dollars a-year.

